# Ethyl 6-*r*-(2-chlorophenyl)-2-oxo-4-phenyl­cyclohex-3-ene-1-*t*-carboxylate

**DOI:** 10.1107/S1600536808043055

**Published:** 2008-12-20

**Authors:** N. Anuradha, A. Thiruvalluvar, K. Pandiarajan, C. Yuvaraj

**Affiliations:** a PG Research Department of Physics, Rajah Serfoji Government College (Autonomous), Thanjavur 613 005, Tamil Nadu, India; b Department of Chemistry, Annamalai University, Annamalai Nagar 608 002, Tamil Nadu, India

## Abstract

In the title mol­ecule, C_21_H_19_ClO_3_, the cyclo­hexene ring adopts an envelope conformation, with all substituents equatorial. The plane through its five coplanar atoms makes dihedral angles of 12.75 (14) and 74.16 (8)° with the phenyl and benzene rings, respectively. The dihedral angle between the latter two rings is 81.73 (12)°. Inter­molecular C—H⋯O hydrogen bonds and intra­molecular C—H⋯Cl contacts are found in the crystal structure; a weak C—H⋯π inter­action is also present.

## Related literature

For synthetic applications and biological activities, see: Cokcer *et al.* (1995 ▶); Friedrich *et al.* (2006 ▶); Pandey *et al.*(2004 ▶); Rebacz *et al.* (2007 ▶). For related crystal structures, see: Guzei (2004 ▶); Shishkina *et al.* (2002 ▶).
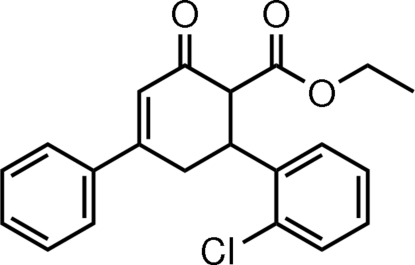


## Experimental

### 

#### Crystal data

C_21_H_19_ClO_3_
*M* *_r_* = 354.81
Triclinic, 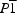

*a* = 8.0115 (3) Å
*b* = 11.3525 (4) Å
*c* = 11.6386 (4) Å
α = 62.261 (2)°
β = 77.975 (2)°
γ = 75.297 (2)°
*V* = 901.16 (6) Å^3^
*Z* = 2
Mo *K*α radiation
μ = 0.23 mm^−1^
*T* = 293 (2) K
0.25 × 0.20 × 0.20 mm

#### Data collection

Bruker Kappa APEXII CCD diffractometer
Absorption correction: multi-scan (*SADABS*; Bruker, 2004 ▶) *T*_min_ = 0.945, *T*_max_ = 0.956
17066 measured reflections
3885 independent reflections
2825 reflections with *I* > 2σ(*I*)
*R*_int_ = 0.028

#### Refinement

*R*[*F* ^2^ > 2σ(*F* ^2^)] = 0.045
*wR*(*F* ^2^) = 0.137
*S* = 1.05
3885 reflections
227 parameters
H-atom parameters constrained
Δρ_max_ = 0.53 e Å^−3^
Δρ_min_ = −0.39 e Å^−3^

### 

Data collection: *APEX2* (Bruker, 2004 ▶); cell refinement: *SAINT-NT* (Bruker, 2004 ▶); data reduction: *SAINT-NT*; program(s) used to solve structure: *SHELXS97* (Sheldrick, 2008 ▶); program(s) used to refine structure: *SHELXL97* (Sheldrick, 2008 ▶); molecular graphics: *ORTEP-3* (Farrugia, 1997 ▶); software used to prepare material for publication: *PLATON* (Spek, 2003 ▶).

## Supplementary Material

Crystal structure: contains datablocks global, I. DOI: 10.1107/S1600536808043055/wn2298sup1.cif

Structure factors: contains datablocks I. DOI: 10.1107/S1600536808043055/wn2298Isup2.hkl

Additional supplementary materials:  crystallographic information; 3D view; checkCIF report

## Figures and Tables

**Table 1:** Hydrogen-bond geometry (Å, °)

*D*—H⋯*A*	*D*—H	H⋯*A*	*D*⋯*A*	*D*—H⋯*A*
C17—H17*A*⋯*Cg*1^i^	0.97	2.89	3.538 (3)	125
C5—H5⋯Cl2	0.98	2.56	3.0843 (19)	114
C33—H33⋯O16^ii^	0.93	2.58	3.296 (3)	134
C56—H56⋯O16^iii^	0.93	2.45	3.308 (3)	154

Symmetry codes: (i) 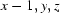; (ii) 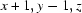; (iii) 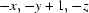. *Cg*1 is the centroid of the C51–C56 ring.

**Fig. 1.**
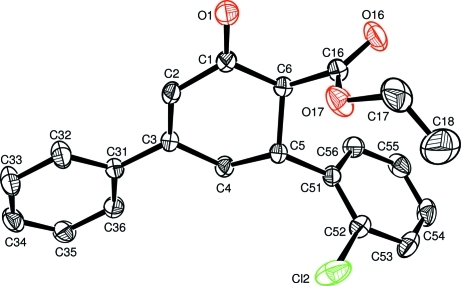
The molecular structure of the title compound, showing the atom-numbering scheme and displacement ellipsoids drawn at the 30% probability level. H atoms have been omitted for clarity.

**Fig. 2.**
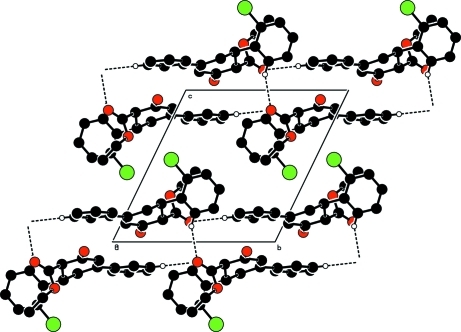
The packing of the title compound, viewed down the a axis. Dashed lines indicate hydrogen bonds. H atoms not involved in hydrogen bonding have been omitted.

## supplementary crystallographic information

### Comment

Cyclohex-2-enone derivatives exhibit antifungal (Friedrich *et al.*, 2006) and anticancer (Rebacz *et al.*,  2007) activities. Cyclohex-2-enones have also been used in organic synthesis (Cokcer *et al.*,  1995; Pandey *et al.*,  2004).

Guzei (2004) has reported a crystal structure determination of 4,4,6,6-tetraphenylcyclohex-2-en-1-one, wherein the cyclohexenone ring is in a half-chair conformation. Shishkina *et al.* (2002) have reported the molecular structures of chloro-, methoxy- and methoxyphenyl-substituted (1R)-arylidene-p-(4-menthen)-3-ones. The cyclohexenone ring in these compounds has a sofa conformation.

The present X-ray diffraction study was undertaken to determine how the conformation of the system is affected by the substitution of a phenyl group at position 4, chlorophenyl group at position 6 and ethoxycarbonyl group at position 1 of the cyclohexenone ring.

In the title molecule, C_21_H_19_ClO_3_, (Fig. 1), the cyclohexene ring adopts an envelope conformation, with all substituents equatorial. The plane through the five coplanar atoms C1/C2/C3/C4/C6 makes dihedral angles of 12.75 (14)° and 74.16 (8)° with the phenyl and benzene rings, respectively. The dihedral angle between the phenyl and benzene rings is 81.73 (12)°. C33—H33···O16(1 + *x*, -1 + *y*, *z*) and C56—H56···O16(-*x*, 1 - *y*, -*z*) intermolecular hydrogen bonds and a C5—H5···Cl2 intramolecular contact are found in the crystal structure. Furthermore, a C17—H17A···π(-1 + *x*, *y*, *z*) interaction involving the benzene (C51—C56) ring is also found (Fig. 2, Table 1).

### Experimental

A mixture of *o*-chlorobenzylideneacetophenone (3.65 g, 0.015 mol), ethyl acetoacetate (2 ml, 0.015 mol) and sodium ethoxide (1 g, 0.015 mol) in absolute alcohol (50 ml) was refluxed for 14 h. After cooling, the reaction mixture was neutralized with 0.1 N HCl. It was than extracted with diethyl ether (3x20 ml). The organic layer was dried over anhydrous sodium sulfate, filtered and the solvents removed by rotary vacuum evaporation. A solid mass was obtained which was recrystallized from ethanol. Yield 2.22 g (61%).

### Refinement

H atoms were positioned geometrically and allowed to ride on their parent atoms, with Csp^2^—H = 0.93, C(methyl)—H = 0.96, C(methylene)—H = 0.97 and C(methine)—H = 0.98 Å; *U*_iso_(H) = k*U*_eq_(C), where k = 1.5 for methyl and 1.2 for all other H atoms.

### Crystal data

**Table:**

C_21_H_19_ClO_3_	*Z* = 2
*M**_r_* = 354.81	*F*(000) = 372
Triclinic, *P*1	*D*_x_ = 1.308 Mg m^−^^3^
Hall symbol: -P 1	Melting point: 389 K
*a* = 8.0115 (3) Å	Mo *K*α radiation, λ = 0.71073 Å
*b* = 11.3525 (4) Å	Cell parameters from 6137 reflections
*c* = 11.6386 (4) Å	θ = 2.2–26.9°
α = 62.261 (2)°	µ = 0.23 mm^−^^1^
β = 77.975 (2)°	*T* = 293 K
γ = 75.297 (2)°	Prism, yellow
*V* = 901.16 (6) Å^3^	0.25 × 0.20 × 0.20 mm

### Data collection

**Table:**

Bruker Kappa APEXII CCD diffractometer	3885 independent reflections
Radiation source: fine-focus sealed tube	2825 reflections with *I* > 2σ(*I*)
graphite	*R*_int_ = 0.028
ω and φ scan	θ_max_ = 27.0°, θ_min_ = 2.2°
Absorption correction: multi-scan (*SADABS*; Bruker, 2004)	*h* = −9→10
*T*_min_ = 0.945, *T*_max_ = 0.956	*k* = −14→14
17066 measured reflections	*l* = −14→14

### Refinement

**Table:**

Refinement on *F*^2^	Primary atom site location: structure-invariant direct methods
Least-squares matrix: full	Secondary atom site location: difference Fourier map
*R*[*F*^2^ > 2σ(*F*^2^)] = 0.045	Hydrogen site location: inferred from neighbouring sites
*wR*(*F*^2^) = 0.137	H-atom parameters constrained
*S* = 1.05	*w* = 1/[σ^2^(*F*_o_^2^) + (0.0591*P*)^2^ + 0.4414*P*] where *P* = (*F*_o_^2^ + 2*F*_c_^2^)/3
3885 reflections	(Δ/σ)_max_ = 0.001
227 parameters	Δρ_max_ = 0.53 e Å^−^^3^
0 restraints	Δρ_min_ = −0.39 e Å^−^^3^

### Special details

**Table:**

Geometry. Bond distances, angles *etc*. have been calculated using the rounded fractional coordinates. All su's are estimated from the variances of the (full) variance-covariance matrix. The cell e.s.d.'s are taken into account in the estimation of distances, angles and torsion angles
Refinement. Refinement of *F*^2^ against ALL reflections. The weighted *R*-factor *wR* and goodness of fit *S* are based on *F*^2^, conventional *R*-factors *R* are based on *F*, with *F* set to zero for negative *F*^2^. The threshold expression of *F*^2^ > 2σ(*F*^2^) is used only for calculating *R*-factors(gt) *etc*. and is not relevant to the choice of reflections for refinement. *R*-factors based on *F*^2^ are statistically about twice as large as those based on *F*, and *R*- factors based on ALL data will be even larger.

### Fractional atomic coordinates and isotropic or equivalent isotropic displacement parameters (Å^2^)

**Table:**

	*x*	*y*	*z*	*U*_iso_*/*U*_eq_	
Cl2	0.13654 (11)	0.11707 (7)	0.54241 (6)	0.0761 (3)	
O1	−0.1208 (2)	0.14508 (16)	0.06258 (18)	0.0571 (6)	
O16	−0.2286 (2)	0.41679 (16)	0.13239 (17)	0.0681 (6)	
O17	−0.2157 (2)	0.21683 (16)	0.30479 (16)	0.0604 (5)	
C1	0.0078 (3)	0.13601 (19)	0.10828 (19)	0.0377 (6)	
C2	0.1564 (3)	0.02735 (19)	0.12187 (19)	0.0384 (6)	
C3	0.3039 (2)	0.01717 (17)	0.16536 (17)	0.0335 (5)	
C4	0.3214 (2)	0.11953 (19)	0.20808 (19)	0.0371 (6)	
C5	0.1463 (2)	0.18685 (17)	0.25162 (17)	0.0323 (5)	
C6	0.0221 (2)	0.24349 (17)	0.14675 (18)	0.0342 (5)	
C16	−0.1553 (3)	0.30400 (19)	0.1907 (2)	0.0410 (6)	
C17	−0.3782 (3)	0.2616 (3)	0.3678 (3)	0.0766 (10)	
C18	−0.3479 (5)	0.3014 (5)	0.4614 (4)	0.127 (2)	
C31	0.4544 (2)	−0.09227 (18)	0.17178 (18)	0.0349 (5)	
C32	0.4368 (3)	−0.2036 (2)	0.1583 (3)	0.0515 (8)	
C33	0.5756 (3)	−0.3071 (2)	0.1670 (3)	0.0587 (8)	
C34	0.7342 (3)	−0.3016 (2)	0.1883 (2)	0.0517 (7)	
C35	0.7554 (3)	−0.1921 (2)	0.1997 (2)	0.0491 (7)	
C36	0.6165 (3)	−0.0874 (2)	0.1915 (2)	0.0439 (7)	
C51	0.1649 (2)	0.29528 (18)	0.28709 (18)	0.0341 (5)	
C52	0.1578 (3)	0.2746 (2)	0.4147 (2)	0.0420 (6)	
C53	0.1702 (3)	0.3765 (3)	0.4449 (2)	0.0546 (8)	
C54	0.1925 (3)	0.5008 (2)	0.3465 (3)	0.0570 (9)	
C55	0.2043 (3)	0.5233 (2)	0.2197 (3)	0.0527 (8)	
C56	0.1904 (3)	0.42222 (19)	0.1901 (2)	0.0435 (6)	
H2	0.14847	−0.03916	0.09893	0.0461*	
H4A	0.37937	0.18852	0.13632	0.0445*	
H4B	0.39318	0.07510	0.27967	0.0445*	
H5	0.09611	0.11693	0.33016	0.0388*	
H6	0.06963	0.31586	0.06916	0.0410*	
H17A	−0.44593	0.33752	0.30241	0.0921*	
H17B	−0.44441	0.18881	0.41165	0.0921*	
H18A	−0.27789	0.22713	0.52462	0.1908*	
H18B	−0.45689	0.32657	0.50465	0.1908*	
H18C	−0.28870	0.37710	0.41723	0.1908*	
H32	0.32983	−0.20837	0.14306	0.0618*	
H33	0.56122	−0.38122	0.15832	0.0704*	
H34	0.82717	−0.37205	0.19504	0.0620*	
H35	0.86358	−0.18757	0.21314	0.0589*	
H36	0.63254	−0.01326	0.19924	0.0527*	
H53	0.16326	0.36044	0.53153	0.0656*	
H54	0.19970	0.56973	0.36623	0.0684*	
H55	0.22178	0.60725	0.15288	0.0632*	
H56	0.19836	0.43932	0.10300	0.0523*	

### Atomic displacement parameters (Å^2^)

**Table:**

	*U*^11^	*U*^22^	*U*^33^	*U*^12^	*U*^13^	*U*^23^
Cl2	0.1257 (7)	0.0703 (4)	0.0378 (3)	−0.0459 (4)	−0.0124 (3)	−0.0126 (3)
O1	0.0451 (9)	0.0615 (10)	0.0857 (12)	0.0041 (7)	−0.0264 (8)	−0.0483 (9)
O16	0.0714 (12)	0.0474 (9)	0.0641 (11)	0.0224 (8)	−0.0139 (9)	−0.0204 (8)
O17	0.0408 (9)	0.0560 (9)	0.0612 (10)	0.0038 (7)	0.0040 (7)	−0.0166 (8)
C1	0.0378 (10)	0.0388 (10)	0.0428 (10)	−0.0026 (8)	−0.0093 (8)	−0.0231 (8)
C2	0.0408 (11)	0.0365 (9)	0.0479 (11)	−0.0026 (8)	−0.0088 (8)	−0.0271 (8)
C3	0.0362 (10)	0.0312 (8)	0.0358 (9)	−0.0033 (7)	−0.0041 (7)	−0.0180 (7)
C4	0.0353 (10)	0.0368 (9)	0.0462 (10)	−0.0036 (8)	−0.0086 (8)	−0.0237 (8)
C5	0.0359 (9)	0.0286 (8)	0.0349 (9)	−0.0042 (7)	−0.0051 (7)	−0.0162 (7)
C6	0.0368 (10)	0.0312 (8)	0.0367 (9)	−0.0030 (7)	−0.0073 (7)	−0.0168 (7)
C16	0.0422 (11)	0.0374 (10)	0.0465 (11)	0.0027 (8)	−0.0119 (9)	−0.0229 (9)
C17	0.0411 (14)	0.097 (2)	0.0765 (19)	0.0071 (13)	0.0046 (12)	−0.0396 (17)
C18	0.080 (2)	0.208 (5)	0.092 (3)	0.017 (3)	−0.005 (2)	−0.088 (3)
C31	0.0368 (10)	0.0316 (9)	0.0360 (9)	−0.0018 (7)	−0.0048 (7)	−0.0163 (7)
C32	0.0427 (12)	0.0403 (11)	0.0781 (16)	−0.0003 (9)	−0.0125 (10)	−0.0323 (11)
C33	0.0542 (14)	0.0395 (11)	0.0866 (18)	0.0027 (10)	−0.0109 (12)	−0.0352 (12)
C34	0.0449 (12)	0.0451 (11)	0.0546 (13)	0.0100 (9)	−0.0069 (10)	−0.0215 (10)
C35	0.0363 (11)	0.0574 (13)	0.0534 (12)	0.0014 (9)	−0.0091 (9)	−0.0272 (10)
C36	0.0409 (11)	0.0464 (11)	0.0502 (12)	−0.0031 (9)	−0.0081 (9)	−0.0270 (10)
C51	0.0318 (9)	0.0336 (9)	0.0412 (10)	−0.0022 (7)	−0.0060 (7)	−0.0209 (8)
C52	0.0449 (11)	0.0455 (11)	0.0420 (10)	−0.0098 (9)	−0.0032 (8)	−0.0240 (9)
C53	0.0567 (14)	0.0699 (15)	0.0593 (14)	−0.0141 (11)	−0.0016 (11)	−0.0468 (13)
C54	0.0540 (14)	0.0514 (13)	0.0883 (18)	−0.0058 (10)	−0.0114 (12)	−0.0494 (13)
C55	0.0541 (13)	0.0330 (10)	0.0732 (16)	−0.0078 (9)	−0.0194 (11)	−0.0200 (10)
C56	0.0511 (12)	0.0365 (10)	0.0449 (11)	−0.0096 (9)	−0.0104 (9)	−0.0163 (9)

### Geometric parameters (Å, °)

**Table:**

Cl2—C52	1.735 (2)	C52—C53	1.387 (4)
O1—C1	1.216 (3)	C53—C54	1.369 (4)
O16—C16	1.192 (3)	C54—C55	1.362 (4)
O17—C16	1.315 (3)	C55—C56	1.377 (4)
O17—C17	1.451 (4)	C2—H2	0.9300
C1—C2	1.454 (3)	C4—H4A	0.9700
C1—C6	1.518 (3)	C4—H4B	0.9700
C2—C3	1.338 (3)	C5—H5	0.9800
C3—C4	1.506 (3)	C6—H6	0.9800
C3—C31	1.481 (3)	C17—H17A	0.9700
C4—C5	1.526 (3)	C17—H17B	0.9700
C5—C6	1.531 (2)	C18—H18A	0.9600
C5—C51	1.517 (3)	C18—H18B	0.9600
C6—C16	1.511 (3)	C18—H18C	0.9600
C17—C18	1.440 (6)	C32—H32	0.9300
C31—C32	1.388 (3)	C33—H33	0.9300
C31—C36	1.384 (3)	C34—H34	0.9300
C32—C33	1.379 (4)	C35—H35	0.9300
C33—C34	1.366 (4)	C36—H36	0.9300
C34—C35	1.365 (3)	C53—H53	0.9300
C35—C36	1.388 (3)	C54—H54	0.9300
C51—C52	1.383 (3)	C55—H55	0.9300
C51—C56	1.390 (3)	C56—H56	0.9300
			
Cl2···O17^i^	3.303 (2)	C36···H4A	3.0700
Cl2···H5	2.5600	C52···H17B^viii^	3.0800
O1···O17	3.181 (3)	C53···H18B^viii^	3.0600
O1···C36^ii^	3.386 (3)	C55···H17A^viii^	3.0800
O16···C56^iii^	3.308 (3)	C55···H34^iv^	2.9700
O16···C33^iv^	3.296 (3)	C56···H4A	2.9700
O17···O1	3.181 (3)	C56···H6	2.6700
O17···C51	3.329 (2)	H2···C32	2.5600
O17···Cl2^i^	3.303 (2)	H2···H32	2.0100
O1···H2^v^	2.7300	H2···O1^v^	2.7300
O1···H32^v^	2.9200	H4A···C36	3.0700
O1···H36^ii^	2.7400	H4A···C56	2.9700
O1···H55^iii^	2.8200	H4A···H36	2.5700
O16···H56^iii^	2.4500	H4B···C36	2.6400
O16···H33^iv^	2.5800	H4B···H36	2.1700
O16···H17A	2.3400	H5···Cl2	2.5600
O17···H5	2.4800	H5···O17	2.4800
C1···C34^vi^	3.561 (3)	H5···C2	2.9500
C1···C35^vi^	3.537 (3)	H6···C56	2.6700
C2···C36^vi^	3.554 (3)	H6···H56	2.1400
C2···C35^vi^	3.325 (3)	H17A···O16	2.3400
C16···C56	3.368 (4)	H17A···C55^ii^	3.0800
C17···C53^ii^	3.597 (4)	H17B···C52^ii^	3.0800
C31···C31^vi^	3.560 (3)	H18B···C53^ii^	3.0600
C33···O16^vii^	3.296 (3)	H18C···C16	3.0300
C34···C1^vi^	3.561 (3)	H32···C2	2.6100
C35···C1^vi^	3.537 (3)	H32···H2	2.0100
C35···C2^vi^	3.325 (3)	H32···H55^x^	2.4100
C36···O1^viii^	3.386 (3)	H32···O1^v^	2.9200
C36···C2^vi^	3.554 (3)	H33···O16^vii^	2.5800
C51···O17	3.329 (2)	H34···C55^vii^	2.9700
C53···C17^viii^	3.597 (4)	H36···O1^viii^	2.7400
C56···C16	3.368 (4)	H36···C4	2.5700
C56···O16^iii^	3.308 (3)	H36···H4A	2.5700
C2···H5	2.9500	H36···H4B	2.1700
C2···H32	2.6100	H53···C35^ix^	2.9200
C4···H36	2.5700	H55···H32^xi^	2.4100
C6···H56	2.7300	H55···O1^iii^	2.8200
C16···H18C	3.0300	H56···C6	2.7300
C32···H2	2.5600	H56···H6	2.1400
C35···H53^ix^	2.9200	H56···O16^iii^	2.4500
C36···H4B	2.6400		
			
C16—O17—C17	118.4 (2)	C3—C4—H4B	109.00
O1—C1—C2	122.0 (2)	C5—C4—H4A	109.00
O1—C1—C6	120.5 (2)	C5—C4—H4B	109.00
C2—C1—C6	117.5 (2)	H4A—C4—H4B	108.00
C1—C2—C3	124.1 (2)	C4—C5—H5	108.00
C2—C3—C4	120.14 (18)	C6—C5—H5	108.00
C2—C3—C31	121.88 (19)	C51—C5—H5	108.00
C4—C3—C31	117.98 (15)	C1—C6—H6	108.00
C3—C4—C5	112.45 (15)	C5—C6—H6	108.00
C4—C5—C6	110.44 (15)	C16—C6—H6	108.00
C4—C5—C51	111.92 (15)	O17—C17—H17A	109.00
C6—C5—C51	111.38 (16)	O17—C17—H17B	109.00
C1—C6—C5	111.37 (17)	C18—C17—H17A	109.00
C1—C6—C16	110.36 (17)	C18—C17—H17B	109.00
C5—C6—C16	111.41 (16)	H17A—C17—H17B	108.00
O16—C16—O17	124.3 (2)	C17—C18—H18A	109.00
O16—C16—C6	124.9 (2)	C17—C18—H18B	109.00
O17—C16—C6	110.76 (19)	C17—C18—H18C	109.00
O17—C17—C18	111.0 (3)	H18A—C18—H18B	109.00
C3—C31—C32	120.76 (18)	H18A—C18—H18C	109.00
C3—C31—C36	121.43 (19)	H18B—C18—H18C	109.00
C32—C31—C36	117.8 (2)	C31—C32—H32	119.00
C31—C32—C33	121.0 (2)	C33—C32—H32	120.00
C32—C33—C34	120.5 (2)	C32—C33—H33	120.00
C33—C34—C35	119.6 (2)	C34—C33—H33	120.00
C34—C35—C36	120.5 (2)	C33—C34—H34	120.00
C31—C36—C35	120.7 (2)	C35—C34—H34	120.00
C5—C51—C52	122.81 (18)	C34—C35—H35	120.00
C5—C51—C56	120.49 (17)	C36—C35—H35	120.00
C52—C51—C56	116.7 (2)	C31—C36—H36	120.00
Cl2—C52—C51	120.13 (18)	C35—C36—H36	120.00
Cl2—C52—C53	118.07 (16)	C52—C53—H53	120.00
C51—C52—C53	121.8 (2)	C54—C53—H53	120.00
C52—C53—C54	119.7 (2)	C53—C54—H54	120.00
C53—C54—C55	119.9 (3)	C55—C54—H54	120.00
C54—C55—C56	120.2 (3)	C54—C55—H55	120.00
C51—C56—C55	121.7 (2)	C56—C55—H55	120.00
C1—C2—H2	118.00	C51—C56—H56	119.00
C3—C2—H2	118.00	C55—C56—H56	119.00
C3—C4—H4A	109.00		
			
C17—O17—C16—O16	−3.1 (4)	C6—C5—C51—C52	134.2 (2)
C17—O17—C16—C6	174.6 (2)	C6—C5—C51—C56	−45.8 (2)
C16—O17—C17—C18	−96.6 (3)	C1—C6—C16—O16	−111.0 (3)
O1—C1—C2—C3	176.5 (2)	C1—C6—C16—O17	71.4 (2)
C6—C1—C2—C3	−0.3 (3)	C5—C6—C16—O16	124.8 (2)
O1—C1—C6—C5	155.26 (19)	C5—C6—C16—O17	−52.9 (2)
O1—C1—C6—C16	31.0 (3)	C3—C31—C32—C33	−178.6 (2)
C2—C1—C6—C5	−27.9 (2)	C36—C31—C32—C33	1.3 (4)
C2—C1—C6—C16	−152.18 (18)	C3—C31—C36—C35	178.79 (18)
C1—C2—C3—C4	2.1 (3)	C32—C31—C36—C35	−1.1 (3)
C1—C2—C3—C31	−177.22 (18)	C31—C32—C33—C34	−0.4 (4)
C2—C3—C4—C5	24.9 (2)	C32—C33—C34—C35	−0.7 (4)
C31—C3—C4—C5	−155.82 (16)	C33—C34—C35—C36	0.9 (3)
C2—C3—C31—C32	−13.2 (3)	C34—C35—C36—C31	0.1 (3)
C2—C3—C31—C36	166.89 (19)	C5—C51—C52—Cl2	3.0 (3)
C4—C3—C31—C32	167.5 (2)	C5—C51—C52—C53	−178.1 (2)
C4—C3—C31—C36	−12.4 (3)	C56—C51—C52—Cl2	−176.99 (18)
C3—C4—C5—C6	−52.1 (2)	C56—C51—C52—C53	1.9 (3)
C3—C4—C5—C51	−176.77 (15)	C5—C51—C56—C55	178.7 (2)
C4—C5—C6—C1	53.2 (2)	C52—C51—C56—C55	−1.3 (3)
C4—C5—C6—C16	176.93 (17)	Cl2—C52—C53—C54	178.0 (2)
C51—C5—C6—C1	178.25 (15)	C51—C52—C53—C54	−0.9 (4)
C51—C5—C6—C16	−58.1 (2)	C52—C53—C54—C55	−0.7 (4)
C4—C5—C51—C52	−101.6 (2)	C53—C54—C55—C56	1.3 (4)
C4—C5—C51—C56	78.3 (2)	C54—C55—C56—C51	−0.3 (4)

Symmetry codes: (i) −*x*, −*y*, −*z*+1; (ii) *x*−1, *y*, *z*; (iii) −*x*, −*y*+1, −*z*; (iv) *x*−1, *y*+1, *z*; (v) −*x*, −*y*, −*z*; (vi) −*x*+1, −*y*, −*z*; (vii) *x*+1, *y*−1, *z*; (viii) *x*+1, *y*, *z*; (ix) −*x*+1, −*y*, −*z*+1; (x) *x*, *y*−1, *z*; (xi) *x*, *y*+1, *z*.

### Hydrogen-bond geometry (Å, °)

**Table:**

*D*—H···*A*	*D*—H	H···*A*	*D*···*A*	*D*—H···*A*
C17—H17A···Cg1^ii^	0.97	2.89	3.538 (3)	125
C5—H5···Cl2	0.98	2.56	3.0843 (19)	114
C33—H33···O16^vii^	0.93	2.58	3.296 (3)	134
C56—H56···O16^iii^	0.93	2.45	3.308 (3)	154

Symmetry codes: (ii) *x*−1, *y*, *z*; (vii) *x*+1, *y*−1, *z*; (iii) −*x*, −*y*+1, −*z*.
